# Clinical validation of an electronic hand hygiene surveillance system

**DOI:** 10.1186/cc14164

**Published:** 2015-03-16

**Authors:** P Levin, R Razon, MJ Cohen, CL Sprung, S Benenson

**Affiliations:** 1 Hadassah Hebrew University Medical Center, Jerusalem, Israel

## Introduction

Good hand hygiene (HH) is critical to infection control in the ICU. Electronic HH surveillance systems are purported to improve HH practices. Such a system was recently trialed in our ICU. The system is based on radiofrequency transponders in three locations: bracelets worn by ICU personnel; on all HH product dispensers; and above each patient's bed. By correlating input from these three sources the system detects whether HH was performed before and after each patient contact. In the event that HH is not performed, the bracelet alerts the user (by vibration) in real time. This study represents a clinical validation of the system.

## Methods

ICU staff (nurses and physicians) were followed by a trained observer over 60-minute periods. Each movement and contact during the period was documented. HH opportunities were determined according to WHO criteria and actual HH performance recorded. Observer and electronic data were compared for number of opportunities, HH performance and compliance. A satisfaction questionnaire was distributed to all users. Paired Student's *t *test was used for comparison of the observer and electronic data.

## Results

Observations were made over 56 time periods that included 836 HH opportunities and 485 occasions when HH was performed. The observer recorded 10.9 ± 7.6 HH opportunities/hour compared with 6.8 ± 6.9 for the electronic system (*P *< 0.001). HH performance occurred on 8.7 ± 3.9 occasions/hour versus 6.0 ± 3.1 occasions/hour as recorded by the electronic system (*P *< 0.001). Overall HH compliance was 62.5 ± 17.7% versus 57.5 ± 21.0% respectively (*P *= 0.523). On comparison of specific observation periods, there was poor correlation between compliance as recorded by the observer and electronic system (*r *= 0.03, *P *= 0.915). Satisfaction questionnaires were completed by 41 personnel. Satisfaction with the system was low or very low for 21/41 (61%). System inaccuracy (either bracelet alerts without cause, or lack of bracelet alerts when HH was required) was the most common reason for dissatisfaction (31/41, 76%), followed by physical discomfort from the bracelet (18/41, 44%).

## Conclusion

The electronic HH system consistently underestimated both HH opportunities and HH performance. The main reason for dissatisfaction with the system was inaccuracy of bracelet alerts. These data suggest that for an electronic system to be accepted by ICU staff, it has to be highly accurate and comfortable for the user.

